# Design, methodology, and baseline of whole city-million scale children and adolescents myopia survey (CAMS) in Wenzhou, China

**DOI:** 10.1186/s40662-021-00255-1

**Published:** 2021-08-19

**Authors:** Liangde Xu, Youyuan Zhuang, Guosi Zhang, Yunlong Ma, Jian Yuan, Changseng Tu, MiaoMiao Li, Wencan Wang, Yaru Zhang, Xiaoyan Lu, Jing Li, Xinting Liu, Zhengbo Xue, Meng Zhou, Jie Sun, Jinhua Bao, Ming Li, Fan Lu, Hong Wang, Jianzhong Su, Jia Qu

**Affiliations:** 1 School of Ophthalmology and Optometry and Eye Hospital, Wenzhou Medical University, Wenzhou, 325027 China; 2 State Key Laboratory of Ophthalmology, Optometry and Visual Science, Wenzhou, 325027 China; 3 Institute of Biomedical Big Data, Wenzhou Medical University, Wenzhou, 325027 China; 4 National Clinical Research Center for Ocular Disease, Wenzhou, 325027 China; 5 College of Bioinformatics Science and Technology, Harbin Medical University, Harbin, 150081 People’s Republic of China

**Keywords:** Baseline, Vision screening, Myopia prevention and control, Population-based

## Abstract

**Background:**

Myopia is the most common visual impairment in children and adolescents worldwide. This study described an economical and effective population-based screening pipeline and performed the project of a million scale children and adolescents myopia survey (CAMS), which will shed light on the further study of myopia from the level of epidemiology and precision medicine.

**Methods:**

We developed a novel population-based screening pattern, an intelligent screening process and internet-based information transmission and analysis system to carry out the survey consisting of school children in Wenzhou, China. The examination items include unaided distance visual acuity, presenting distance visual acuity, and non-cycloplegic autorefraction. Myopia and high myopia were defined as spherical equivalent (SE) ≤ − 1.00 diopters (D) and SE ≤ − 6.00 D, respectively. Next, the reports of the vision checking were automatically sent to parents and the related departments. The CAMS project will be done two to four times annually with the support of the government. An online eyesight status information management system (OESIMS) was developed to construct comprehensive and efficient electronic vision health records (EVHRs) for myopia information inquiry, risk pre-warning, and further study.

**Results:**

The CAMS completed the first-round of screening within 30 days for 99.41% of Wenzhou students from districts and counties, in June 2019. A total of 1,060,925 participants were eligible for CAMS and 1,054,251 (99.37% participation rate) were selected through data quality control, which comprised 1305 schools, and 580,609, 251,050 and 170,967 elementary, middle, and high school students. The mean age of participants was 12.21 ± 3.32 years (6–20 years), the female-to-male ratio was 0.82. The prevalence of myopia in elementary, middle, and high school students was 38.16%, 77.52%, and 84.00%, respectively, and the high myopia incidence was 0.95%, 6.90%, and 12.98%.

**Conclusions:**

The CAMS standardized myopia screening model involves automating large-scale information collection, data transmission, data analysis and early warning, thereby supporting myopia prevention and control. The entire survey reduced 90% of staff, cost, and time consumption compared with previous surveys. This will provide new insights for decision support for public health intervention.

**Supplementary Information:**

The online version contains supplementary material available at 10.1186/s40662-021-00255-1.

## Background

Refractive error is one of the most common eye ailments in the world [1]. In recent years, the prevalence of myopia is rising rapidly [2–7]. Individuals with high myopia have a higher risk of the development of permanent visual impairment or blindness due to retinal detachment, macular degeneration, cataract, and glaucoma [8–15]. The potential global productivity loss caused by vision impairment of uncorrected myopia was estimated at US$244,000 million in 2015 [16]. Based on existing trends, myopia is expected to affect about 49.8% of the world's population by the year 2050 [17].

In the last decade, a series of cross-sectional or prospective studies in refractive errors have been performed worldwide, such as in Asia [18–31], Europe [2, 32–40], America [41–45], Oceania [46–49], and Africa [50–54]. These studies provided essential information on the epidemiology of major ocular disorders and visual impairment among various countries and ethnic groups. In addition, these studies have indicated that the prevalence of refractive errors varies considerably depending on age, sex, ethnicity, and geographical environment. From a global perspective, myopia has a higher prevalence rate in East Asian countries (including China, Japan, South Korea, and Singapore) [55–64].

The myopia prevalence rate in China is higher than in other East Asian countries (for example, India and Nepal) [5, 27, 65–67]. A nationwide survey (2014) from China reported that the prevalence of myopia had reached 80% among students who had completed secondary education [68]. What is more disturbing is that vision impairment in children impacts their economic status and employment in adult life through influencing their psychological, social, and educational development [69]. Currently, there are vast numbers of cross-sectional refractive data for school-aged children, but longitudinal studies in this age group are relatively fewer and small-scale, even though investigating the incidence of myopia is of critical importance for formulating appropriate public health policies.

In the last year, the coronavirus disease (COVID-19) had led to an unprecedented global pandemic. To contain COVID-19, strict containment measures were imposed internationally, including social-distancing regulations, limited outdoor, school closures and switching in-person education to online home-based learning [70, 71]. With the implementation of these measures, citizens spent more time using digital devices for entertainment and education. This rapid increase in digital screen time may potentially lead to a rise of myopia rates worldwide, especially in Asia [72, 73]. Therefore, investigating the prevalence of myopia before and after the COVID-19 epidemic will help us understand the effect of factors such as digital devices and near activity on myopia prevalence.

The children and adolescents myopia survey (CAMS) is a population-based, city-wide, prospective cross-sectional survey undertaken to meet ocular health requirements of school-aged children and adolescents for the entire city of Wenzhou. This study screened 1.06 million students at over 1300 filtered schools. The initial cross-sectional survey was conducted in Jun 2019, while the subsequent follow-up study will last for ten years conducted at least twice annually. By December 2020, the follow-up survey had been performed four times. The main objective of this study was to determine the prevalence of myopia and high myopia. Secondary goals included recording the dynamic changes of myopia prevalence over the next decade and determining the prevalence of the other refractive errors. Furthermore, the CAMS includes a questionnaire survey to examine the living environment, eating habits, reading time, outdoor time and digital screen time, and the posture of near work. In addition, whole-exome sequencing and ophthalmic examination have been performed for high myopia students. The project results will contribute to public health intervention for eye health and clarify the molecular biological mechanism in myopia progression.

## Methods

### Ethics

The CAMS conformed to the principles of the Helsinki Declaration and has received ethics approval from the Eye Hospital of Wenzhou Medical University.

### Study area and population

**Fig. 1:**
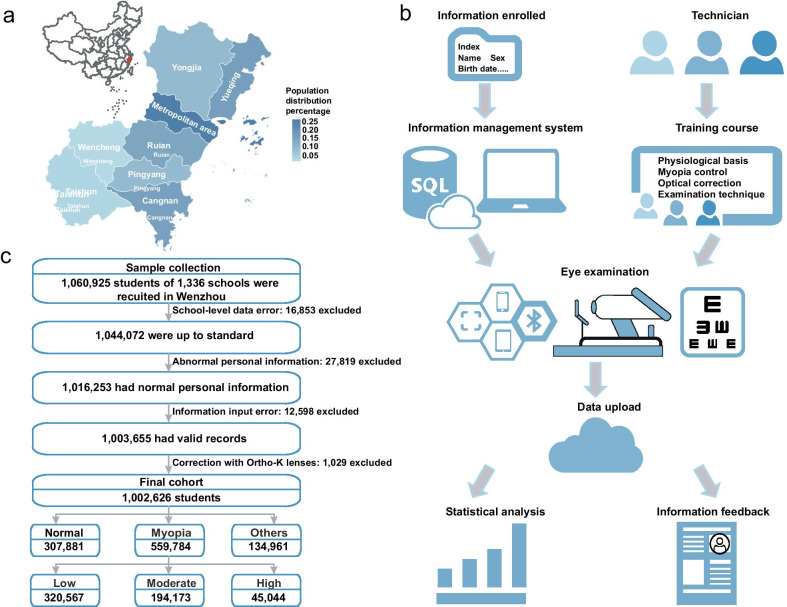
Flowchart of the CAMS. **a** Location of the CAMS and regional population percentage of Wenzhou. **b** Framework of the CAMS. **c** Quality control, showing the participants available for analysis in the baseline

Wenzhou is located in southeast China (Fig. 1a), covering an area of nearly 11,613 km^2^, with 9.12 million permanent residents [74]. According to the demographic data (2019) released by the Wenzhou Statistical Bureau, about 6.55 million urban residents and 2.74 million rural residents have been living in the city [74]. There are four districts (Lucheng, Longwan, Ouhai, and Dongtou), two country-level cities (Ruian and Yueqing), and five counties (Yongjia, Pingyang, Cangnan, Wencheng, and Taishun). Wenzhou is one of the most developed cities in Zhejiang Province, whose gross domestic product (GDP) is 6606.1 billion RMB (in 2019), ranking 3rd in Zhejiang Province and placed 30th overall in China [75, 76].

### Study design and sampling process

The CAMS was a population-based, prospective cross-sectional survey initiated in May 2019, and funded by the Wenzhou government. This study aimed to enroll all school-aged children and adolescents from the elementary, middle, and high schools in the entire area of Wenzhou. Most notably, the CAMS is more than just vision screening; it is a combination of modernized vision screening, vision information management system, and electronic vison health records (EVHRs) (Fig. 1b). The study sought to attain four specific research objectives: (1) To determine the prevalence of myopia and other refractive errors in Wenzhou children and adolescents; (2) To record the dynamic changes of myopia prevalence over the next decade; (3) To publicize the knowledge of eye health care at the same time as the survey (such as playing a series of short cartoons about eye health knowledge in the waiting room, and handing out leaflets about eye health care); and (4) To delay the progress of myopia through early detection and intervention (CAMS provided the vision screening results and recommendations for participants on the EVHR, which is available for students and their parents to query). To date, the follow-up surveys have been completed four times, and the scale and intensity will remain the same as the initial vision screening in the coming 9 years.

### Study committee

Led by the Wenzhou government and Wenzhou Education Bureau, a study committee was set up to organize, coordinate, and supervise the myopia survey. The office of the study committee was located at the Biomedical Big Data Center of Wenzhou Medical University.

### Recruitment strategies

Prior to the commencement of the study, the Wenzhou Education Bureau notified the principals of each school about the CAMS. Subsequently, the principals conveyed the notification to the school health nurses and school doctors. Meanwhile, the education bureau sent text messages about the CAMS to all elementary, middle, and high school students. In addition, posters and radio broadcasts were displayed throughout the schools to further promote the study. It was gratifying to observe that no parents or students refused to participate in the CAMS, except for those with very specific personal reasons, such as sick leave and personal leave. The participation rate (the number of participants/the total number invited to participate) was 99.37% (1,054,251/1,060,925).

### Information management system and electronic vision health records

To promote the efficiency of vision screening, it is necessary to establish a modernized information management system. In the preparation phase, we set up an online eyesight status information management system (OESIMS) for children and adolescents (see Additional file 1 Supplementary Materials 1 for details). The OESIMS was developed to upload, store, and manage vision screening data. With the support and assistance of the Wenzhou Education Bureau, the students’ general information (including the full name, student ID, ID number, class, grade, school type, school name, and administrative division) had been imported into the system by our team before the investigation. It is worth mentioning that the system supports real-time data uploads from an autorefractor via wireless networks or Bluetooth. Moreover, based on the OESIMS, an EVHR system was created to allow parents to query all visual examination results through their mobile phones.

### Screening personnel

Vision screening personnel consisted school health nurses, health care physicians (school doctors), and volunteers from graduate students in Optometry. The guidelines proposed earlier for school vision screening indicated that school health nurses are capable screeners in a majority of countries [77]. Therefore, it is reasonable to incorporate school health nurses as screeners. To guarantee that the vision screening project could be carried out smoothly and professionally, the local government issued a policy to train one capable screener per 250 students. The training mission was delegated to the Eye Hospital of Wenzhou Medical University by the Wenzhou government. Subsequently, the eye hospital organized senior optometrists to train the targeted training groups, including school nurses and health care physicians. The entire training comprised four hours of theoretical training and four hours of practical training, and was carried out twice a year. The locations for centralized training were local hospitals of each district and county of Wenzhou. The exams were carried out immediately after training, and only by passing the exam could a screener be regarded as a capable screener.

### Data collection procedures

The data acquisition process was as follows: (1) contact elementary, middle, and high schools; (2) schedule the survey days and organize screening; (3) choose suitable examination sites (usually the classroom); (4) distribute the brochures with personal information and a quick response (QR) code to each student through teachers; (5) propagate knowledge of eye health care; (6) perform ocular examination and upload data; and (7) download data from the OESIMS for further analysis and decision-making.

### Ophthalmic examination

#### a) Distance visual acuity examination

Distance visual acuity (DVA) was assessed monocularly with a Chinese standard logarithm visual acuity E chart (GB 11533-2011) in an illuminated cabinet (WSVC-100, Wenzhou, China). If participants' prescription was worn, both uncorrected distance visual acuity (UDVA) and presenting distance visual acuity (PDVA) were measured. Otherwise, only UDVA was measured. Visual acuity (VA) examination was carried out at a distance of 5 meters from the chart [78–80]. Participants who failed to identify any optotypes were asked to approach the E chart until they could identify the optotypes. Subsequently, VA was recorded as (0.1 × distance)/5′ [81]. If any optotypes were still unrecognized, VA was assessed as counting fingers, hand motions, light perception, or no light perception. The results of VA examination were temporarily recorded in the students' brochures, waiting for input into the system.

#### b) Non-cycloplegic autorefraction

Every participant underwent a non-cycloplegic autorefraction after the VA measurement. Prior to examination, the screeners used a digital tablet to scan each participant’s QR code to enter the individual editing module of OESIMS and filled in the VA test results. Non-cycloplegic autorefraction tests were conducted using “Goaleye RM-9000 (Shenzhen Aist Industrial Co., Ltd., China; Former name: GoldEye RM-9000) autorefractors”. Each eye was measured three times. Thereafter, the results of autorefraction were automatically uploaded into the corresponding OESIMS editing module via wireless or Bluetooth.

### Definition for visual impairment and refraction

#### Visual impairment

According to the World Health Organization (WHO) 2003 criteria, no visual impairment was defined as PDVA ≥ 6/12, mild impairment as 6/18 ≤ PDVA < 6/12, moderate visual impairment as 6/60 ≤ PDVA < 6/8, and severe visual impairment as 3/60 ≤ PDVA < 6/60 [82].

### Refraction

The spherical equivalent (SE) reported in the present study were non-cycloplegic SE. Since non-cycloplegic autorefraction is likely to overestimate myopia prevalence in the young population [83, 84], we adopted a stricter definition of myopia. Likely myopia was defined as − 1.00 D < SE ≤ − 0.50 D in at least one eye, and myopia was defined as SE ≤ − 1.00 D in at least one eye [83]. It was further subdivided for analysis into low myopia (− 3.00 D < SE ≤ − 1.00 D), moderate myopia (− 6.00 D < SE ≤ − 3.00 D), and high myopia (SE ≤ − 6.00 D) [85]. Hyperopia was defined as SE ≥ + 2.00 D in any eye [84, 86]. Astigmatism was defined as minus cylinders ≤ − 0.75 D in any eye [86]. Anisometropia was defined as SE difference between both eyes based on 1.50 D [87].

### Data quality control and analysis

To ensure the reliability and accuracy of the domestic autorefractor (Goaleye RM-9000), 449 individuals aged from 9 to 18 years (12.3 ± 2.1 years) chosen randomly from Wenzhou city were measured by three autorefractors in random sequence, including Goaleye RM-9000 and two reputable autorefractors (Topcon RM-800 (Tokyo, Japan) and Nidek AR-1 (Nagoya, Japan)) [88]. Then, a senior optometrist performed a subjective refraction for them by phoropter [88]. To monitor the validity of the data, each school carried out the self-examination by simple random sampling of 5% of the students. In the meantime, the local health department randomly selected 5% of the students from 5% of the schools randomly selected, to conduct ocular examinations for further verification.

The data downloaded from OESIMS was filtered by quality control procedures before analysis (Fig. 1c). The statistical analyses were performed by SPSS software (version 20.0). The prevalence of myopia and high myopia was calculated by the worse eye. As for categorical variables, Pearson chi-squared tests were performed. All confidence intervals were 95% intervals.

## Results

### Establishing the advanced information management system

After evaluating the effectiveness of the project process, on the basis of Wilcoxon signed ranks test, we found that the differences of SE between Goaleye RM-9000 and subjective refraction were even smaller compared with that between Topcon RM-800/Nidek AR-1 and subjective refraction [88]. Therefore, based on the Goaleye RM-9000, and combining high-precision technology with the vision screening, the OESIMS for the large-scale myopia survey of children and adolescents was developed by our team in the early stages. The system, generating sole index numbers and unique QR codes for each student, was developed to upload, store, and manage vision screening data. By scanning the QR code distributed by the teachers in advance, screeners could quickly enter the individual editing module of OESIMS and input the results of the VA test. More than that, the OESIMS also supports real-time data upload from the autorefractors via wireless networks or Bluetooth. These functions greatly facilitated data collection and uploading, reduced the workload, and saved time.

### Baseline information

**Fig. 2:**
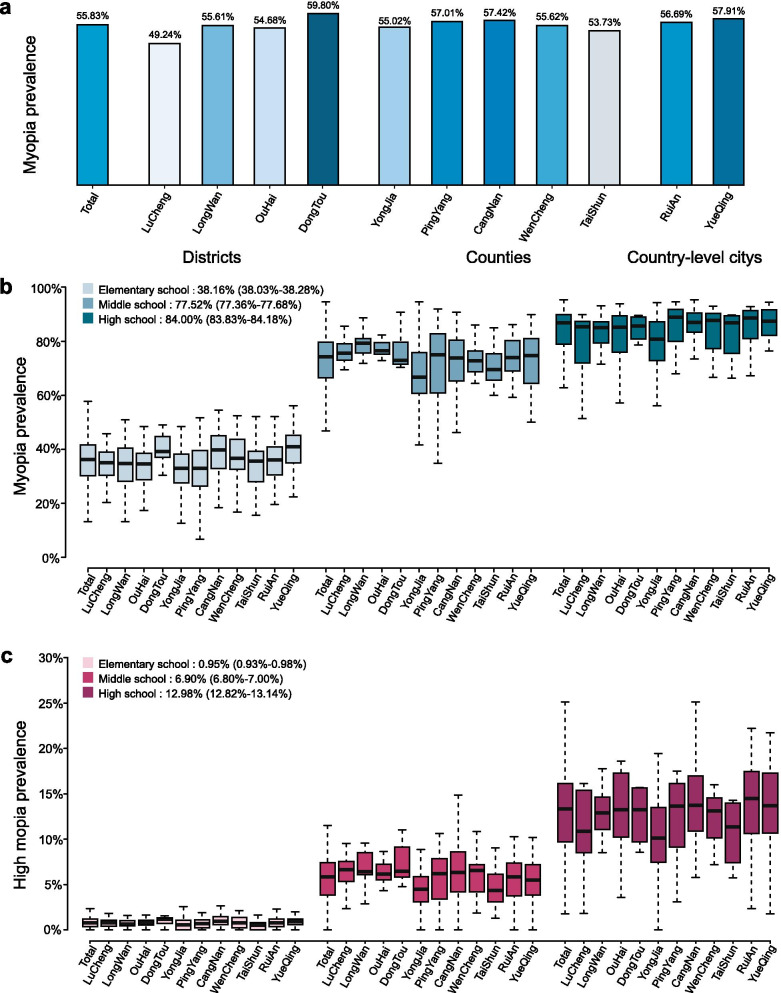
Myopia prevalence of different regions in Wenzhou. **a** A total of eleven counties, districts, and cities. **b** Myopia prevalence. **c** High myopia prevalence

The baseline characteristics are presented as follows: of 1,060,925 individuals eligible for myopia screening, 1,054,251 took part in the study (99.37% participation rate). With data quality control, 1,002,626 (95.10%) were included for further analysis. The average myopia rate of each county was close to 55.83% (n = 559,784), as shown in Fig. 2a. In addition, the survey shows significant differences in the rate of myopia among children in urban and rural areas (Table 1). Myopia (SE ≤ − 0.50 D) prevalence was 58.64% (95% CI 58.53%–58.75%) in urban schools, and 50.30% (95% CI 50.30%–50.54%) in rural schools (*P* < 0.0001). The prevalence of high myopia was 5.05% (95% CI 5.00%–5.10%) in urban schools, and 3.12% (95% CI, 3.03%–3.20%) in rural schools (*P* < 0.0001). Figure 2b and c show the distribution of myopia and high myopia rates in elementary, middle, and high schools in different areas of Wenzhou, with myopia rates reaching 38.16%, 77.52%, and 84.00%, respectively.

**Table 1:** Distribution of myopia and high myopia in different regions

	Count	SE, mean (SD) (D)	Likely myopia (95% CI)	Myopia (95% CI)	High myopia (95% CI)
Region					
Urban area	777,779	− 1.93 (2.16)	6.82% (6.77%–6.88%)	58.64% (58.53%–58.75%)	5.05% (5.00%–5.10%)
Rural area	160,275	− 1.5 (1.96)	7.55% (7.42%–7.68%)	50.30% (50.05%–50.54%)	3.12% (3.03%–3.20%)
Unknown	64,572	− 0.89 (1.61)	7.26% (7.06%–7.46%)	35.80% (35.43%–36.17%)	1.22% (1.14%–1.31%)
Location					
Lucheng^a^	98,953	− 1.49 (2.02)	6.87% (6.71%–7.03%)	49.24% (48.93%–49.55%)	3.35% (3.24%–3.47%)
Longwan^a^	71,287	− 1.79 (2.14)	7.24% (7.05%–7.43%)	55.61% (55.24%–55.97%)	4.67% (4.52%–4.83%)
Ouhai^a^	80,964	− 1.8 (2.17)	6.68% (6.51%–6.86%)	54.68% (54.34%–55.03%)	4.91% (4.77%–5.06%)
Dongtou^a^	13,927	− 1.98 (2.19)	6.74% (6.33%–7.16%)	59.80% (58.98%–60.61%)	5.39% (5.02%–5.77%)
Yongjia^b^	98,243	− 1.78 (2.09)	6.46% (6.31%–6.62%)	55.02% (54.71%–55.34%)	4.22% (4.09%–4.34%)
Pingyang^b^	96,957	− 1.92 (2.15)	6.35% (6.19%–6.50%)	57.01% (56.70%–57.32%)	4.75% (4.62%–4.89%)
Cangnan^b^	165,083	− 1.88 (2.13)	7.26% (7.14%–7.39%)	57.42% (57.18%–57.65%)	4.81% (4.71%–4.91%)
Wencheng^b^	24,214	− 1.75 (2.06)	7.99% (7.65%–8.33%)	55.62% (54.99%–56.24%)	4.20% (3.94%–4.45%)
Taishun^b^	32,335	− 1.65 (1.99)	7.56% (7.28%–7.85%)	53.73% (53.19%–54.27%)	3.43% (3.23%–3.62%)
Ruian^c^	150,508	− 1.85 (2.14)	6.86% (6.74%–6.99%)	56.69% (56.44%–56.94%)	4.76% (4.65%–4.87%)
Yueqing^c^	170,155	− 1.82 (2.12)	7.25% (7.13%–7.38%)	57.91% (57.67%–58.14%)	4.51% (4.42%–4.61%)

*SE* spherical equivalent; *SD* standard deviation; *CI* confidence interval

^a^District

^b^Country

^c^Country-level city

**Fig. 3:**
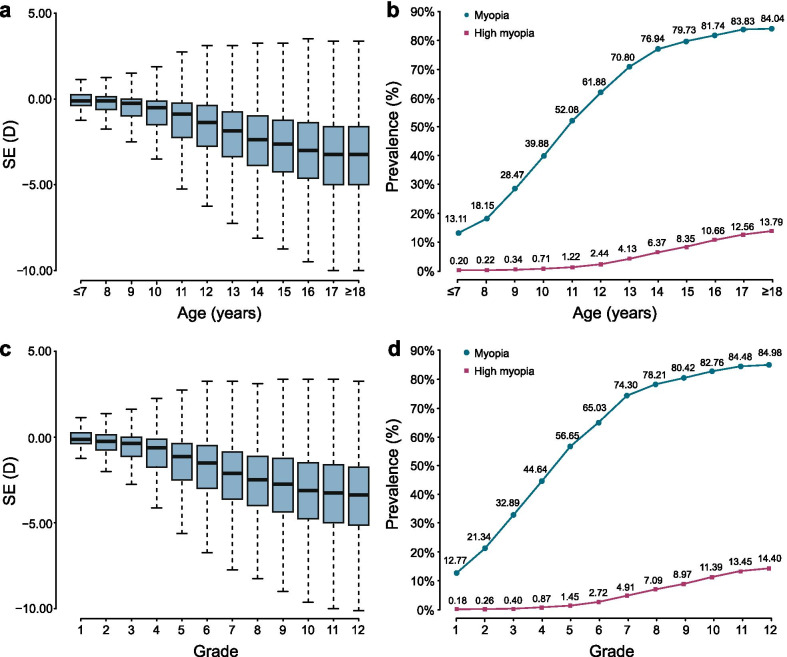
Spherical equivalent (SE) distribution and myopia prevalence for children and adolescents. **a** SE distribution for school students aged 6 to 20 years. **b** Myopia prevalence for school students aged 6 to 20 years. **c** SE distribution for school students with different grades. **d** Myopia prevalence for school students of different grades

Over half of the participants were males (54.98%, n = 551,251). The average age was 12.21 ± 3.32 years (ranging from 6 to 20 years). In addition, the prevalence of myopia between males and females exhibited significant differences (53.29% for males vs. 58.94% for females, *P* < 0.0001), as shown in Table 2. We analyzed the VA status of students of different ages. The mean SE of participants and the prevalence of myopia and high myopia are reported in Tables 2 and 3. We found that the myopia prevalence in elementary and middle school students continued to increase with age (Fig. 3), and the rate of high myopia rose exponentially with age. In terms of VA of students in different grades, we hypothesized that the education phase is another influencing factor associated with myopia. The prevalence of myopia was 38.16% (95% CI 38.03%–38.28%) in elementary schools, 77.52% (95% CI 77.36%–77.68%) in middle schools, and 84.00% (95% CI 83.83%–84.18%) in high schools. High myopia prevalence in elementary, middle, and high schools were 0.95% (95% CI 0.93%–0.98%), 6.90% (95% CI 6.80%–7.00%), and 12.98% (95% CI 12.82%–13.14%), respectively. Statistical tests showed that there was a significant difference between grades for both myopia and high myopia (*P* < 0.0001).

**Table 2:** Distribution of myopia and high myopia in different genders and ages

	Count	SE, Mean (SD) (D)	Likely myopia (95% CI)	Myopia (95% CI)	High myopia (95% CI)
Gender					
Male	551,251	− 1.72 (2.11)	6.72% (6.65%–6.79%)	53.29% (53.16%–53.42%)	4.38% (4.32%–4.43%)
Female	451,375	− 1.89 (2.12)	7.27% (7.19%–7.34%)	58.94% (58.79%–59.08%)	4.63% (4.57%–4.69%)
Age (years)					
≤ 7^a^	67,256	− 0.14 (0.97)	6.01% (5.83%–6.19%)	13.11% (12.85%–13.36%)	0.20% (0.16%–0.23%)
8	99,877	− 0.29 (1.06)	6.92% (6.76%–7.07%)	18.15% (17.91%–18.38%)	0.22% (0.19%–0.25%)
9	94,796	− 0.60 (1.24)	8.44% (8.26%–8.62%)	28.47% (28.18%–28.76%)	0.34% (0.30%–0.37%)
10	95,303	− 0.95 (1.42)	9.35% (9.17%–9.54%)	39.88% (39.57%–40.19%)	0.71% (0.65%–0.76%)
11	95,587	− 1.36 (1.61)	9.60% (9.41%–9.78%)	52.08% (51.76%–52.39%)	1.22% (1.15%–1.29%)
12	93,219	− 1.76 (1.78)	8.68% (8.50%–8.86%)	61.88% (61.56%–62.19%)	2.24% (2.15%–2.34%)
13	89,646	− 2.22 (1.95)	7.54% (7.37%–7.71%)	70.80% (70.50%–71.10%)	4.13% (4.00%–4.26%)
14	87,081	− 2.63 (2.08)	6.19% (6.03%–6.35%)	76.94% (76.66%–77.22%)	6.37% (6.21%–6.53%)
15	84,692	− 2.91 (2.17)	5.19% (5.04%–5.34%)	79.73% (79.46%–80.00%)	8.35% (8.17%–8.54%)
16	65,200	− 3.16 (2.28)	4.53% (4.37%–4.69%)	81.74% (81.44%–82.03%)	10.66% (10.42%–10.89%)
17	61,339	− 3.38 (2.31)	4.06% (3.90%–4.22%)	83.83% (83.54%–84.12%)	12.56% (12.30%–12.82%)
≥ 18^b^	68,630	− 3.44 (2.37)	3.97% (3.82%–4.12%)	84.04% (83.77%–84.32%)	13.79% (13.53%–14.05%)

*SE* spherical equivalent; *SD* standard deviation; *CI* confidence interval

^a^The minimum age is 6 years old

^b^The maximum age is 20 years old

**Table 3:** Distribution of myopia and high myopia in different educational levels

Education phase	Grade	Count	SE, Mean (SD) (D)	Likely myopia (95% CI)	Myopia (95% CI)	High myopia (95% CI)
Elementary school	1	104,727	− 0.13 (0.97)	5.82% (5.68%–5.96%)	12.77% (12.57%–12.98%)	0.18% (0.16%–0.21%)
	2	98,456	− 0.39 (1.11)	7.54% (7.38%–7.71%)	21.34% (21.08%–21.59%)	0.26% (0.23%–0.29%)
	3	95,606	− 0.72 (1.29)	9.20% (9.02%–9.38%)	32.89% (32.59%–33.19%)	0.40% (0.36%–0.44%)
	4	96,269	− 1.10 (1.48)	9.63% (9.45%–9.82%)	44.64% (44.32%–44.95%)	0.87% (0.81%–0.93%)
	5	94,529	− 1.52 (1.66)	9.43% (9.24%–9.61%)	56.65% (56.34%–56.97%)	1.45% (1.38%–1.53%)
	6	91,022	− 1.91 (1.83)	8.27% (8.09%–8.45%)	65.03% (64.72%–65.34%)	2.72% (2.61%–2.83%)
	Total	580,609	− 0.94 (1.54)	8.27% (8.20%–8.34%)	38.16% (38.03%–38.28%)	0.95% (0.93%–0.98%)
Middle school	7	87,817	− 2.40 (1.99)	7.07% (6.90%–7.24%)	74.30% (74.01%–74.59%)	4.91% (4.77%–5.05%)
	8	86,165	− 2.75 (2.11)	5.85% (5.69%–6.00%)	78.21% (77.93%–78.48%)	7.09% (6.92%–7.26%)
	9	77,068	− 2.99 (2.20)	4.77% (4.62%–4.92%)	80.42% (80.14%–80.70%)	8.97% (8.76%–9.17%)
	Total	251,050	− 2.70 (2.11)	5.94% (5.85%–6.04%)	77.52% (77.36%–77.68%)	6.90% (6.80%–7.00%)
High school	10	61,714	− 3.26 (2.30)	4.32% (4.16%–4.49%)	82.76% (82.47%–83.06%)	11.39% (11.14%–11.64%)
	11	59,858	− 3.46 (2.32)	3.97% (3.82%–4.13%)	84.48% (84.19%–84.77%)	13.45% (13.17%–13.72%)
	12	49,395	− 3.51 (2.38)	3.74% (3.58%–3.91%)	84.98% (84.66%–85.29%)	14.40% (14.09%–14.71%)
	Total	170,967	− 3.40 (2.33)	4.03% (3.94%–4.13%)	84.00% (83.83%–84.18%)	12.98% (12.82%–13.14%)

*SE* spherical equivalent; *SD* standard deviation; *CI* confidence interval

**Fig. 4:**
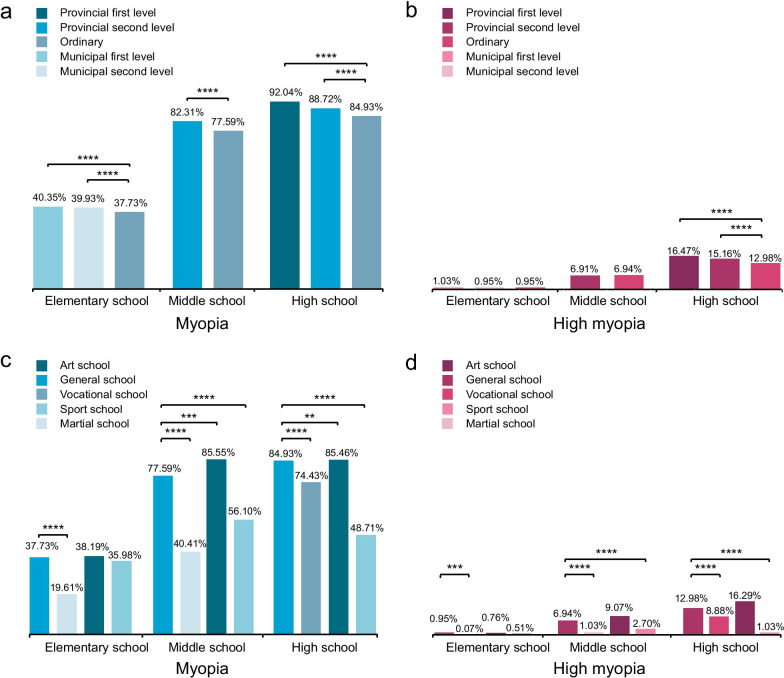
Prevalence of myopia and high myopia in different school levels and school types. **a** & **b** According to school level, elementary, middle, and high schools were divided into provincial first level, provincial second level, ordinary, municipal first level and municipal second level. **c** & **d** According to school type, elementary, middle, and high schools were divided into art school, general school, vocational school, sport school and martial school. Pearson chi-squared test was performed to determine significance between each pair of condition. *P*-value: **P < 0.01, ***P < 0.001, ****P < 0.0001

There were obvious differences in the prevalence of myopia among various types of schools. The myopia rate in key schools was significantly higher than that in general schools (Pearson chi-squared test, *P* < 0.0001). Figure 4a shows that there were significant differences in myopia and high myopia rates between key and non-key schools at different stages. The myopia rate in middle schools at the provincial second level was more than 82%, and that in high schools at the provincial first level was more than 90%. Moreover, in primary school, the myopia rate of martial arts schools was 19.61%, significantly lower than that of general schools (37.73%, *P* < 0.0001) at the same stage (Table 4). In middle and high schools, the myopia rates in martial arts schools, art schools, and sports schools were significantly lower than that in general schools.

**Table 4:** Distribution of myopia and high myopia in different type of schools

Education phase	Type of school	Count	SE, Mean (SD) (D)	Likely myopia (95% CI)	Myopia (95% CI)	High myopia (95% CI)
Elementary school	General school	460,412	− 0.92 (1.54)	8.26% (8.18%–8.34%)	37.73% (37.59%–37.87%)	0.95% (0.92%–0.98%)
	Key school	114,353	− 1.00 (1.56)	8.37% (8.21%–8.53%)	40.11% (39.83%–40.40%)	0.98% (0.93%–1.04%)
	Martial arts school	1433	− 0.40 (1.18)	5.72% (4.52%–6.92%)	19.61% (17.55%–21.66%)	0.07% (0.00%–0.21%)
	Art school	3430	− 0.86 (1.49)	7.81% (6.92%–8.71%)	38.19% (36.57%–39.82%)	0.76% (0.47%–1.05%)
	Sport school	981	− 0.87 (1.47)	8.46% (6.72%–10.20%)	35.98% (32.98%–38.99%)	0.51% (0.06%–0.96%)
Middle school	General school	242,409	− 2.71 (2.11)	5.95% (5.86%–6.05%)	77.59% (77.42%–77.75%)	6.94% (6.84%–7.04%)
	Key school	6511	− 2.83 (2.03)	5.45% (4.90%–6.00%)	82.31% (81.38%–83.23%)	6.91% (6.30%–7.53%)
	Martial arts school	777	− 1.13 (1.64)	7.46% (5.62%–9.31%)	40.41% (36.96%–43.86%)	1.03% (0.32%–1.74%)
	Art school	353	− 3.03 (2.01)	3.12% (1.30%–4.93%)	85.55% (81.88%–89.22%)	9.07% (6.07%–12.06%)
	Sport school	1000	− 1.67 (1.82)	6.50% (4.97%–8.03%)	56.10% (53.02%–59.18%)	2.70% (1.70%–3.70%)
High school	General school	36,202	− 3.48 (2.26)	3.47% (3.28%–3.66%)	84.93% (84.56%–85.29%)	12.98% (12.63%–13.33%)
	Key school	84,077	− 3.78 (2.27)	3.18% (3.07%–3.30%)	89.43% (89.22%–89.63%)	15.44% (15.19%–15.68%)
	Vocational school	49,729	− 2.72 (2.32)	5.83% (5.63%–6.04%)	74.43% (74.04%–74.81%)	8.88% (8.63%–9.13%)
	Art school	571	− 3.49 (2.36)	5.08% (3.28%–6.88%)	85.46% (82.57%–88.36%)	16.29% (13.26%–19.32%)
	Sport school	388	− 1.33 (1.46)	8.51% (5.73%–11.28%)	48.71% (43.74%–53.68%)	1.03% (0.03%–2.04%)

*SE* spherical equivalent; *SD* standard deviation; *CI* confidence interval

The CAMS analyzed the vision and refractive status of children and adolescents in Wenzhou. More than 358,317 school students have not corrected myopia. The percentages of students with undercorrected VA in elementary, junior high, and senior high school were 13.13% (95% CI 12.74%–13.52%), 18.49% (95% CI 18.08%–18.90%), and 15.65% (95% CI 15.16%–16.13%), respectively (Table 5). The percentage of students with uncorrected VA was 68.18% (95% CI 67.94%–68.41%) in elementary schools, 42.29% (95% CI 41.95%–42.63%) in middle schools, and 30.86% (95% CI 30.43%–31.30%) in high schools.

**Table 5:** Percentage of students with corrected, undercorrected and uncorrected visual acuity among different education phases

Type of school	Corrected	Undercorrected	Uncorrected
Elementary school	18.69% (18.31%–19.07%)	13.13% (12.74%–13.52%)	68.18% (67.94%–68.41%)
Junior high school	39.22% (38.87%–39.57%)	18.49% (18.08%–18.90%)	42.29% (41.95%–42.63%)
Senior high school	53.49% (53.13%–53.85%)	15.65% (15.16%–16.13%)	30.86% (30.43%–31.30%)
Total	34.66% (34.44%–34.87%)	15.63% (15.39%–15.87%)	49.71% (49.53%–49.90%)

## Discussion

In this study, we described the design, methodology, and baseline profile of students in the CAMS. During the process, the Wenzhou government, the Wenzhou Education Bureau, and the Eye Hospital of Wenzhou Medical University came together to make this vision screening successful. The myopia survey is the first step of the CAMS project. The next step involves exploring environmental factors and genetic factors associated with myopia. We started a series of follow-up studies in 2020, including whole-exome sequencing and ophthalmic examination for students with high myopia along with diet and lifestyle questionnaires. CAMS strives to facilitate the automation of information collection, cloud data transmission, modular data storage, and intelligent data analysis. Data from the CAMS will not only provide detailed information on the prevalence of myopia/high myopia in school-age children and adolescents aged 6 to 20 years for the entire city of Wenzhou, but also is a powerful aid in devising myopia control strategies for China. The baseline information obtained from vision screening indicated that the current situation of students’ myopia control in Wenzhou is truly grim. There is still much work to be done to improve myopia prevention and control and eye care services.

Our study evaluated the prevalence of myopia in Wenzhou schoolchildren in southeast China. The myopia prevalence was 55.83% (55.73%–55.93%) for the 6- to 20-year-old age group and the SE was myopic (− 3.10 ± 1.91 D). Prior research indicated myopia prevalence in China, ranging from 33.9% in Chongqing (children aged 7 to 13 years) [89], 36.7% in the Chaoyang District of Beijing (children aged 5 to 14 years) [90] to 47.4% in Guangzhou (children grades 1 to 9) [91]. For myopia prevalence in other countries in Asia, the prevalence ranged from 23.8% in north India (children aged 13 to 15 years) [27], 54.05% in Japan (children aged 12 to 14 years) [92], 73.0% in South Korea (children aged 12 to 18 years) [93] to 74.9% in Singapore (children aged 15 to 19 years) [94]. It was particularly worrying that the prevalence of myopia in our research exceeded the previous report in Singapore, reaching 77.54% (children aged 15 to 19 years). In addition, the prevalence of myopia varied greatly among different types of schools (Fig. 4a, b) in our data. In high school, myopia prevalence at key schools was the highest (Fig. 4c, d), while that of the sports school and martial arts school was lower (Fig. 4). Key schools in China usually mean a richer learning environment, with better teacher resources, better electronic equipment resources, compared with other school types. Students at key schools tend to spend more time on reading and completing learning tasks, while students from sports school and martial arts school tend to have more outdoor time and exercise time. This indirectly shows that near work can promote myopia, while outdoor and sports can help to reduce the risk of developing myopia.

In the CAMS project, the first round of the myopia survey for children and adolescents in the whole city was successfully completed in May 2019, indicating that the standardized and procedural process created by our committee can be implemented and promoted nationwide as a model. We have established an elaborate workflow for vision screening through integrating automated technology into traditional vision screening. The workflow has the following advantages.

First, fast screening: With real-time data uploads from an autorefractor via wireless networks or Bluetooth, it takes only 22.5 seconds for each student to complete the whole process, and only 15 min to complete all the examinations of 40 students in a class.

Second, low cost: The costs of each student's examinations are cut by 90%, to as low as 3.5 RMB (0.53 USD) per person. In terms of this large-scale vision screening in millions of students, we put forward two solutions: purchasing services from relevant companies and purchasing equipment for on-campus self-examination by trained school nurses, trained school doctors, and volunteers from graduate students majoring in Optometry. After repeated detailed accounting for the work intensity, manpower, and overall cost of the two methods, we found that the cost of purchasing the service would be 10 RMB (1.52 USD) per student. However, the on-campus self-examination scheme would cost only 3.5 RMB (0.53 USD) per student if the vision screening was conducted twice annually. This average cost included the depreciation of equipment cost of 1.5 RMB (0.23 USD) and a service fee of 2 RMB (0.3 USD) per student. Therefore, our committee introduced this survey model of in-school self-examination, which reduced costs by 90% compared to purchasing the service.

Third, full coverage of the following three aspects: (1) The full coverage of school ages: all students that ranged from the first grade of elementary school to the last year of high school enrolled in the survey. (2) The full coverage of area: each type of school in Wenzhou was included in the survey; only by covering the entire city could we truly understand and evaluate visual impairment and refractive errors. (3) The full coverage of screening frequency: initial vision screening was conducted four times annually, followed by at least two screenings each year. The screenings make it possible for researchers and the government to follow the dynamic process of myopia progression in real time.

Last, convenient system of information management: each child's EVHR was established after data collection. For the parents, the EVHR supports self-service query for eye examination result and provides scientific myopia control suggestions based on their children's vision status (see Additional file 1 Supplementary Materials 2 and 3 for details). For the government, mastering the EVHR is conducive to understanding the current myopia situation in the region, and is an immense help for tracking myopia status and developing public health strategies to effectively reduce the public health burden caused by myopia. For epidemiologists, EVHR provide helpful clues for reducing the incidence and delaying the progress of myopia.

With the CAMS, we carried out training for school doctors and general teachers and promoted the introduction of myopia prevention and control techniques into schools. At present, a total of 4,000 school doctors, school health nurses, and teachers have been trained in myopia-related professional knowledge and skills, and an echelon of personnel and a public network have been formed for myopia surveying, prevention, and control. The CAMS offers a new way to promote public knowledge of eye health, improve the awareness of eye health, and monitor the dynamics of the myopia prevalence rate.

Using cycloplegic autorefraction is a practical necessity in our research. Although cycloplegic autorefraction is an important means to obtain accurate diopters, it is hard to achieve in such a large-scale myopia screening. Time and resources were difficult to coordinate, and parents had great resistance to cycloplegia [80]. Admittedly, cycloplegia is the gold standard for refractive error measurement in young children. However, the application of cycloplegia is also a barrier for large-scale detection. Compared with the pursuit of accurate measurement results, we thought it is more important for more children to have the opportunity to know the status of their vision, which is particularly important for a country like China with a high prevalence of myopia and a large population. Therefore, the study committee decided to adopt a non-cycloplegic refraction.

## Conclusions

In summary, the CAMS is the largest-to-date study that investigated the prevalence of myopia in children and adolescents aged 6 to 20 years in Wenzhou, China. Furthermore, we established the Wenzhou vision screening pattern, which is an integration of modernized vision screening, OESIMS, and EVHR. The pattern possessed the characteristics of practicability, high feasibility, low cost, and modernization. It is hoped that the study can contribute insights for the designing of eye care strategies to alleviate the burden of visual impairment, myopia, and high myopia.

## Supplementary Information

**Additional file 1.** Supplementary Materials.

## Data Availability

The datasets used and/or analyzed during the current study are not publicly available, due to the local government’s policy on non-disclosure of student information.

## Abbreviations

D: Diopters
GDP: Gross domestic product
EVHR: Electronic vision health records
PDVA: Presenting distance visual acuity
OESIMS: Online eyesight status information management system
QR: Quick response
SE: Spherical equivalent
UDVA: Uncorrected distance visual acuity
VA: Visual acuity
CAMS: Child and adolescent myopia survey
WHO: World Health Organization
