# Supplementary material for: Design, methodology, and baseline of whole city-million scale children and adolescents myopia survey (CAMS) in Wenzhou, China
Source: Eye Vis (Lond). 2021 Aug 19;8:31. doi: 10.1186/s40662-021-00255-1 (PMC8373605; doi:10.1186/s40662-021-00255-1)
Supplement: Supplementary file 1 — Additional file 1. Supplementary Materials. [file 40662_2021_255_MOESM1_ESM.docx]

**Additional file 1: Supplymentary metarials 1. Online eyesight status information management system (OESIMS)**

Basic information of each student provided by Wenzhou Education Bureau (including name, sex, birth date, etc) was imported into an online eyesight status— information management system (OESIMS) in advance. OESIMS was based on a dedicated database developed using Microsoft SQL Server 2008 (Microsoft, Redmond, WA, USA). A part of data measured by autorefraction was automatically uploaded into the OESIMS. Another part of data got from distance visual acuity examination were entered by screening personnel into the system. The OESIMS could be accessed at multiple points within the various jurisdictions. In addition, the OESIMS generated sole index numbers and unique QR codes for each student. Screening personnel could scan each subject’s QR code to enter the individual editing module of OESIMS so as to input information more conveniently.

**Additional file 2: Supplymentary metarials 2. Children's vision status**

1.Judgment condition of hyperopia: SE ≥ +2.00 D. The corresponding feedback: Preliminary judgment is hyperopia. Advice to attend the fixed point hospital for further examination and dispensing spectacles.

2.Judgment condition of normal visual acuity: UDVA ≥0.8 Snellen and 0 D≤ SE <+2.00 D. The corresponding feedback: Visual acuity is normal. Please keep it up. reading the myopia control suggestions if needed.

3. Judgment condition of potential risk of developing myopia: UDVA ≥0.8 Snellen and −0.50 D <SE <0.00D (Either eye). The corresponding feedback: Visual acuity is normal, however the spherical equivalent suggests risk of developing myopia. Please pay attention to eye health (reading the myopia control suggestions for details).

4. Judgment condition of uncorrected refractive error: SE ≤ −0.50 D and lack of prescribed spectacles. The corresponding feedback: Preliminary judgment is uncorrected refractive error. Advice to attend the fixed point hospital for further examination and dispensing spectacles. Please pay attention to eye health (reading the myopia control suggestions for details).

5. Judgment condition of under-corrected refractive error: SE ≤ −0.50 D, wearing prescribed spectacles and PDVA < 0.8 Snellen. The corresponding feedback: Preliminary judgment is under-corrected refractive error. Advice to attend the fixed point hospital for further examination and dispensing spectacles. Please pay attention to eye health (reading the myopia control suggestions for details).

6. Judgment condition of likely myopia: −1.00 D < SE ≤ −0.50 D in at least one eye. The corresponding feedback: Preliminary judgment is likely myopia. Advice to attend the fixed point hospital for further examination. Please pay attention to eye health (reading the myopia control suggestions for details).

7. Judgment condition of low myopia: −3.00 D < SE ≤ −1.00 D in at least one eye. The corresponding feedback: Preliminary judgment is low myopia. Please pay attention to eye health (reading the myopia control suggestions for details).

8. Judgment condition of moderate myopia: −6.00 D < SE ≤−3.00 D in at least one eye. The corresponding feedback: Preliminary judgment is moderate myopia. Please pay attention to eye health (reading the myopia control suggestions for details).

9. Judgment condition of high myopia: SE ≤ −6.00 D in at least one eye. The corresponding feedback: Preliminary judgment is high myopia. Individuals with high myopia have a higher risk of the development of permanent visual impairment or blindness due to retinal detachment, macular degeneration, cataract, and glaucoma. Advice to attend the fixed point hospital for further examination. Please pay attention to eye health (reading the myopia control suggestions for details).

**Additional file 3: Supplymentary metarials 3. Scientific myopia control suggestions**

1.Outdoor activity time more than 2 hours per day.

2.Ensuring adequate sleep time (10 hours for elementary school students, 9 hours for middle school students, 8 hours for high school students).

3.The 20-20-20 principle: For every 20 minutes spent staring at a book or computer, people should take a break and look at least 20 feet away for 20 seconds.

4.When using a digital screen, try to choose a digital screen with a larger size and higher resolution as much as possible.

5.Distance to books or digital tablets was more than 30 cm during reading or playing.

6. Distance to TV was less than 2m and/or distance to computer was less than 50 cm.
